# Beaver Tail Liver: A Rare Anatomic Variant

**DOI:** 10.7759/cureus.44579

**Published:** 2023-09-02

**Authors:** Sonia Kapoor, Timothy Williams, Michael Ea, Alejandro Biglione

**Affiliations:** 1 Dr. Kiran C. Patel College of Osteopathic Medicine, Nova Southeastern University, Fort Lauderdale, USA; 2 Internal Medicine, Wellington Regional Medical Center, Wellington, USA

**Keywords:** color flow doppler, internal medicine, radiology, general surgery, beaver tail liver, rare anatomic variants, gastroenterology and hepatology, emergency medicine and trauma

## Abstract

Beaver tail liver is a rare hepatic anatomical variant in which the left hepatic lobe extends into the left upper quadrant and surrounds the spleen. This extension of the left hepatic lobe consists of normal hepatic parenchyma with no functional liver impairment. In trauma cases, however, the extended left hepatic lobe is vulnerable to injury and confused for a splenic injury due to similar echogenicities and densities on ultrasound and CT. It is also misdiagnosed as a splenic subcapsular hematoma, perisplenic hemorrhage, or mass. Usually, the beaver tail liver is encountered incidentally in patients. We present a 67-year-old male with a history of chronic obstructive lung disease, coronary artery disease, myocardial infarction, and aortic valve replacement. The patient was admitted for further evaluation and placed under the Baker Act for attempting to overdose on oxycodone to commit suicide. Initial imaging identified an ill-defined lesion on CT angiography, which raised concerns for potential malignancy of the liver. Ultimately, an MRI of the abdomen ruled out a malignant lesion due to a lack of abnormal contrast enhancement over the circumscribed region. Consequently, further imaging of the liver led to the incidental discovery of the beaver tail liver in this patient. Due to the rarity of this variant, available literature regarding beaver tail liver is limited to several case reports describing it as an incidental finding. This case highlights the rare nature and unique challenges the beaver tail liver presents for emergency medicine physicians, surgeons, and radiologists interpreting imaging studies without knowledge of its existence. It is important to emphasize how the unexpected presence of the left hepatic lobe in the upper left quadrant of the abdomen can lead to misinterpretations in FAST (focused assessment with sonography in trauma) exams and CT scans. Using non-invasive tools, such as color Doppler, is one way to reduce the incorrect diagnosis of hepatic anatomic variants.

## Introduction

Beaver tail liver is a rare hepatic anatomical variant in which the left hepatic lobe extends into the left upper quadrant and surrounds the spleen [1]. The extension of the left hepatic lobe also consists of normal hepatic parenchyma [1]. The beaver tail liver is usually encountered incidentally on abdominal imaging [1]. It is more common in females and involves no functional impairment of the liver [1]. However, in cases of trauma to the left upper quadrant of the abdomen, the extended left hepatic lobe is more susceptible to injury and often confused for a splenic injury due to similar densities and echogenicities on ultrasound and CT [1]. In other instances, it is also misdiagnosed as a splenic subcapsular hematoma or mass [1]. Therefore, providers need to be aware of this variant of hepatic morphology, especially in emergent cases involving abdominal trauma.

## Case presentation

The patient is a 67-year-old male with a past medical history of cirrhosis, chronic obstructive pulmonary disease (COPD), coronary artery disease (CAD), myocardial infarction, and aortic valve replacement. The patient arrived via emergency medical services at Wellington Regional Medical Center after he experienced a syncopal event and was found unconscious with pinpoint pupils at home. After the patient regained consciousness, he admitted to consuming ten 30 milligram tablets of oxycodone due to depression and an attempt to commit suicide. On initial exam, the patient was awake and alert after receiving Narcan en route and at the emergency department (ED). On admission, the patient presented with a heart rate of 107 beats per minute, blood pressure of 156/69 mmHg, and respiration rate of 23 breaths per minute. The urine drug screen was only positive for cannabinoids. The patient was placed under the Baker Act, also known as the Florida Mental Health Act, which led to involuntary hospitalization for examination, provision of crisis services for mental illness, and prevention of self-harm.

Initially, abdominal ultrasound demonstrated a fatty liver, splenomegaly, and status post cholecystectomy. Next, CT angiography of the chest revealed an ill-defined 5.1 x 4.6 cm lesion in segment IV of the liver and splenomegaly. Alpha-fetoprotein tumor marker test was a normal level of 1.4 ng/ml. Further imaging was performed to rule out malignancy. 

**Figure 1:**
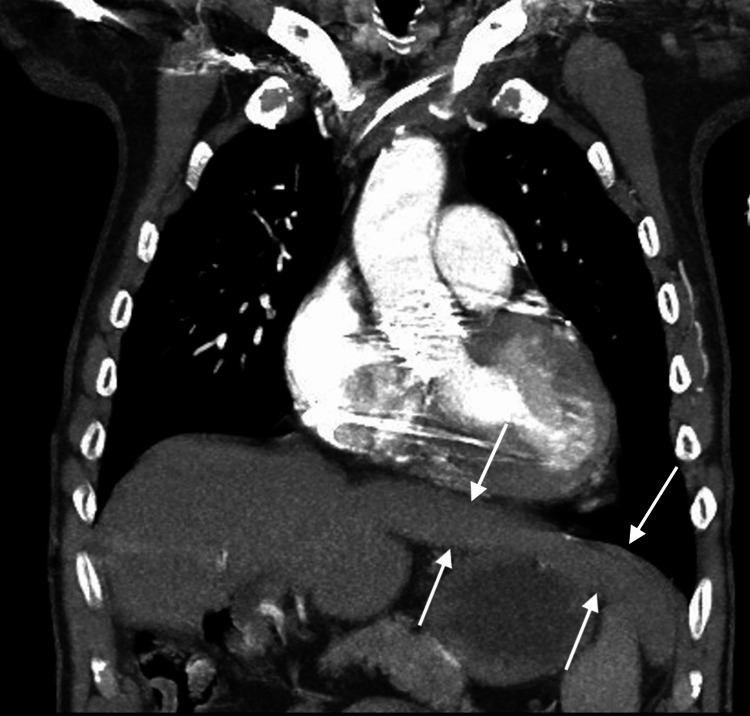
CT angiography of the chest reveals the incidental finding of the beaver tail liver as indicated by the arrows.

**Figure 2:**
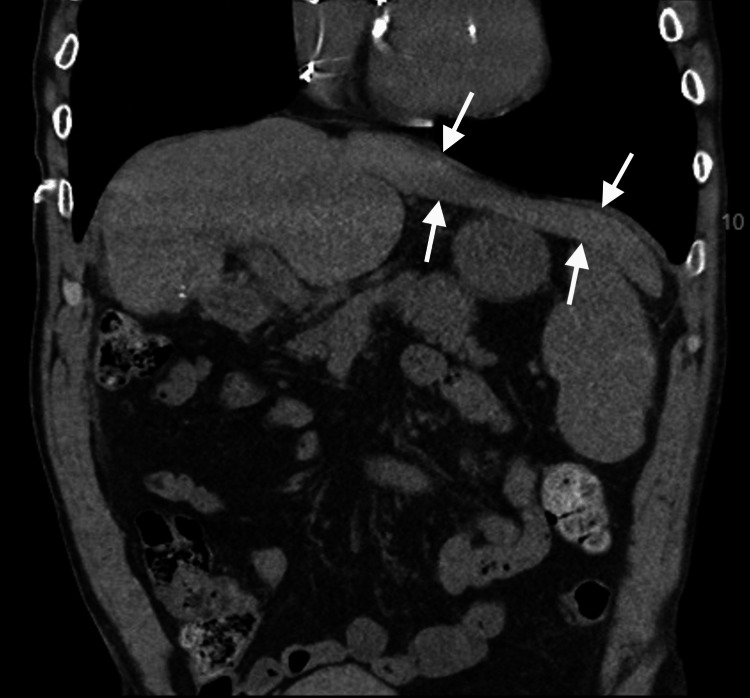
CT of the abdomen shows the incidental finding of the beaver tail liver as indicated by the arrows.

**Figure 3:**
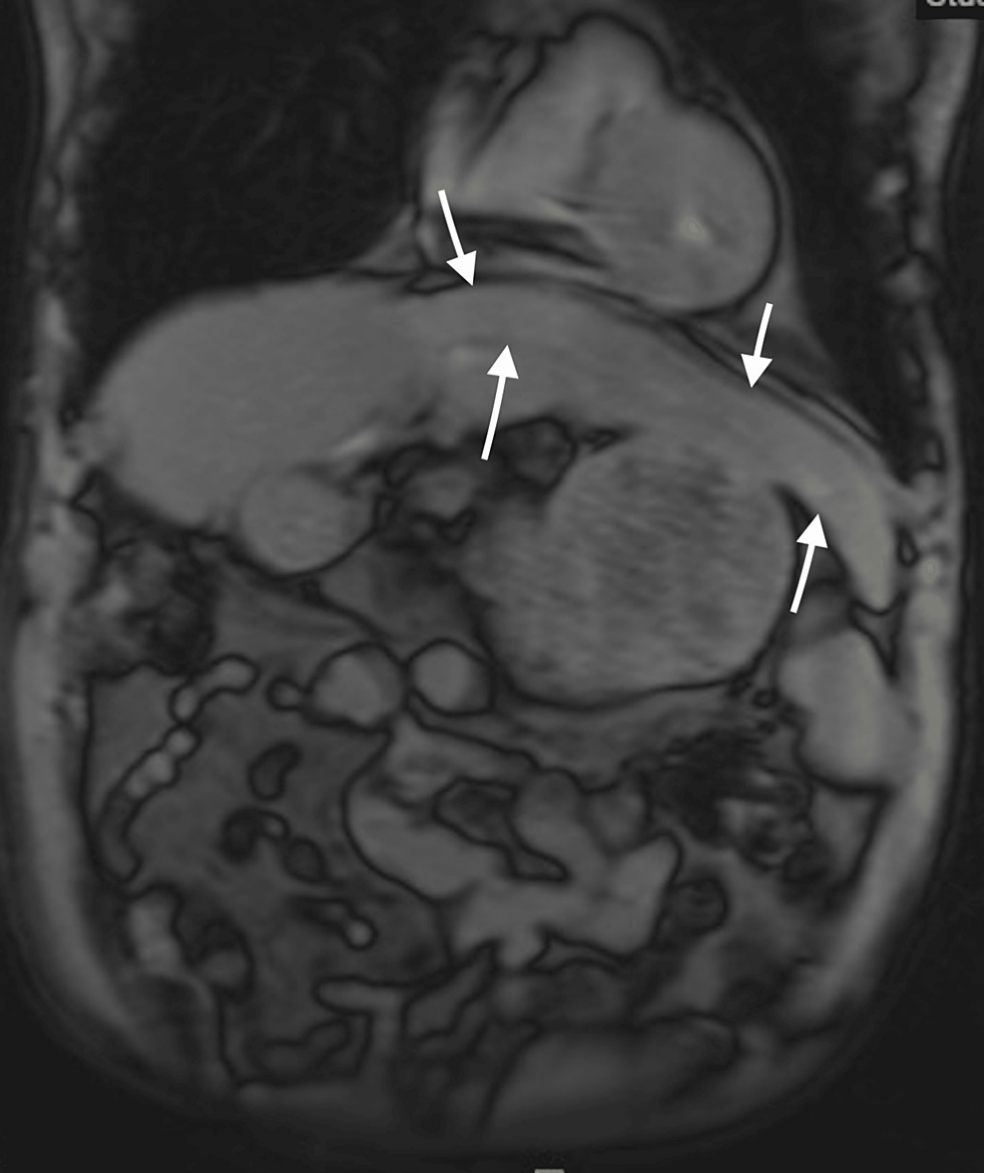
MRI of the abdomen illustrates the incidental finding of the beaver tail liver as indicated by the arrows.

CT of the abdomen showed partial right hepatectomy and a non-enhancing, rounded soft-tissue lesion with peripheral fat attenuation posterior to the hepatic surgical staple line thought to represent focal fat necrosis. Finally, an MRI of the abdomen confirmed a zone of fat necrosis or fatty degeneration over an oval-shaped, circumscribed 3.5 cm region at the surgical site with no abnormal contrast enhancement to suggest a mass lesion. With no mass visualized, interventional radiology did not perform a biopsy. But, additional imaging of the liver led to the incidental discovery of the beaver tail liver (Figures 1-3). The patient was advised to follow up outpatient with gastroenterology. Upon completion of medical management, the patient was discharged to an inpatient psychiatric facility for further mental health care and treatment.

## Discussion

An anatomic variant of the liver is an atypical finding. Hepatic variants include accessory lobes, which are contiguous with the main portion of the liver and consist of normal liver parenchyma [2]. Meanwhile, ectopic lobes are separate from the liver [2]. Riedel’s lobe, a more common hepatic anatomical variant, is a tongue-like inferior extrusion of the anterior border of the right lobe [3]. In comparison, the beaver tail liver is relatively uncommon and presents as a lateral extension of the left lobe of the liver that wraps around the spleen [1]. Due to the rarity of this variant, available literature regarding beaver tail liver is limited to several case reports describing it as an incidental finding. This case highlights the rare nature and unique challenges the beaver tail liver presents for emergency medicine physicians, surgeons, and radiologists interpreting imaging studies without knowledge of its existence. 

In the setting of suspected trauma to the liver or spleen, a routine focused assessment with sonography in trauma (FAST) can be misinterpreted by the unexpected presence of the left hepatic lobe in the left posterior subphrenic space [1,4]. The elongated lobe, which wraps around the spleen, can be confused with a subcapsular hematoma, perisplenic hemorrhage, or splenic mass depending on the echogenicity of the patient’s liver [1,4]. For example, the elongated left lobe of the liver can appear as a hypoechoic, crescent-shaped region between the diaphragm and the spleen, which is similar to a splenic subcapsular hematoma sonographically on FAST exam of the perisplenic window [4]. The transducer can be angled anteriorly or rotated 90 degrees to determine if the potentially elongated left hepatic lobe is contiguous with the right lobe of the liver [4]. However, additional imaging is required if FAST exam findings remain ambiguous. 

On CT scans, the Hounsfield Unit (HU) attenuation of the liver is typically greater than that of the spleen. On average, the spleen measures approximately 40-60 HU while a non-pathological liver measures between 50 and 65 HU [1,5]. However, in patients with mild hepatic steatosis, such as ours, decreased liver attenuation can appear similar to the spleen, which can further confound interpretation [5].

To reduce incorrect diagnoses, color Doppler can be utilized as a non-invasive tool to differentiate between hepatic anatomic variants and the spleen [4]. Color Doppler can help confirm the presence of hepatic and portal veins in the left upper quadrant of the abdomen, leading to the consideration of beaver tail liver [4].

## Conclusions

Beaver tail liver is a rare hepatic anatomic variant that presents with an extension of the left hepatic lobe contacting or wrapping around the spleen. Anatomical variants of the liver, such as the beaver tail liver, can present challenges for emergency medicine physicians, surgeons, and radiologists interpreting imaging studies without knowledge of their existence. Practitioners need to be aware of different methods of differentiating between the liver and the spleen organs to reduce false-positive results on FAST exams and CT scans along with confusion about the anatomy. The elongated left lobe of the liver can be missed if the physician is unfamiliar with its presence and does not actively search for it. It is important to consider hepatic anatomic variants during patient examinations and utilize, for example, different transducer angles during FAST exams and color Doppler to distinguish between the liver and the spleen.
